# Medication Adherence and Blood Pressure Control Among Hypertensive Patients With Coexisting Long-Term Conditions in Primary Care Settings: A Cross-Sectional Analysis: Erratum

**DOI:** 10.1097/MD.0000000000007831

**Published:** 2017-08-11

For the article, “Medication Adherence and Blood Pressure Control Among Hypertensive Patients With Coexisting Long-Term Conditions in Primary Care Settings: A Cross-Sectional Analysis”,^[1]^ which appeared in Volume 95, Issue 20 of *Medicine*, an author requested erratum is being published to acknowledge that a retroactive license has been obtained for use of the Morisky Medication Adherence scale (MMAS-8) used in this article. The captions for all tables should include the following: “Use of the ©MMAS is protected by US copyright laws. Permission for use is required. A license agreement is available from: Donald E. Morisky, MMAS Research (MORISKY) 14725 NE 20th St. Bellevue WA 98007.”

The following references were inadvertently left out from the manuscript as it applies to the MMAS-8 scale:

- Morisky DE, Ang A, Krousel-Wood M, Ward H. Predictive Validity of a Medication Adherence Measure for Hypertension Control. Journal of Clinical Hypertension 2008;10(5):348–354.
- Krousel-Wood MA, Islam T, Webber LS, Re RS, Morisky DE, Muntner P. New Medication Adherence Scale Versus Pharmacy Fill Rates in Seniors with Hypertension. Am J Manag Care 2009;15(1):59–66.
- Morisky DE, DiMatteo MR. Improving the measurement of self-reported medication nonadherence: Final response. J Clin Epidemi 2011;64:258–263. PMID:21144706

The first paragraph in the Methods - Outcome Variables and Covariates should instead read: “Optimal BP control was defined as having a clinical measurement of systolic BP (SBP) <140 mm Hg and diastolic BP (DBP) <90 mm Hg, whereas for patients with diabetes mellitus or chronic kidney diseases, the corresponding threshold was SBP <130 mm Hg and DBP <80 mm Hg. Medication adherence was assessed by the 8-item Morisky Medication Adherence Scale (MMAS-8), which is a commonly used, validated, and self-reported adherence measure of cardiovascular mediations.”
